# Exploring the Crosstalk Between *LMNA* and Splicing Machinery Gene Mutations in Dilated Cardiomyopathy

**DOI:** 10.3389/fgene.2018.00231

**Published:** 2018-07-09

**Authors:** Hind C. Zahr, Diana E. Jaalouk

**Affiliations:** Department of Biology, Faculty of Arts and Sciences, American University of Beirut, Beirut, Lebanon

**Keywords:** lamins, splicing, RNA binding proteins, cardiomyopathies, DCM, RBM20

## Abstract

Mutations in the *LMNA* gene, which encodes for the nuclear lamina proteins lamins A and C, are responsible for a diverse group of diseases known as laminopathies. One type of laminopathy is Dilated Cardiomyopathy (DCM), a heart muscle disease characterized by dilation of the left ventricle and impaired systolic function, often leading to heart failure and sudden cardiac death. *LMNA* is the second most commonly mutated gene in DCM. In addition to *LMNA*, mutations in more than 60 genes have been associated with DCM. The DCM-associated genes encode a variety of proteins including transcription factors, cytoskeletal, Ca^2+^-regulating, ion-channel, desmosomal, sarcomeric, and nuclear-membrane proteins. Another important category among DCM-causing genes emerged upon the identification of DCM-causing mutations in RNA binding motif protein 20 (RBM20), an alternative splicing factor that is chiefly expressed in the heart. In addition to RBM20, several essential splicing factors were validated, by employing mouse knock out models, to be embryonically lethal due to aberrant cardiogenesis. Furthermore, heart-specific deletion of some of these splicing factors was found to result in aberrant splicing of their targets and DCM development. In addition to splicing alterations, advances in next generation sequencing highlighted the association between splice-site mutations in several genes and DCM. This review summarizes *LMNA* mutations and splicing alterations in DCM and discusses how the interaction between *LMNA* and splicing regulators could possibly explain DCM disease mechanisms.

## Introduction

Cardiomyopathies are a diverse group of diseases that affect the heart muscle often rendering it hypertrophied, dilated or rigid. As these diseases progress, the heart becomes weakened and unable to perform its normal mechanical and electrical functions (Maron et al., 2006). Dilated cardiomyopathy (DCM), the most common type of cardiomyopathy, is characterized by dilation of the left or both ventricles and impaired systolic function, in the absence of ischemic coronary artery disease or abnormal pressure or volume loading (Yancy et al., 2013; Pinto et al., 2016). Despite being a rare disease, DCM represents a serious health burden that affects both adults and children, often leading to heart failure and sudden cardiac death (Elliott et al., 2008). Indeed, DCM is one of the leading causes of heart failure and heart transplantation in the world, with a prevalence ranging from 1 case per 2500 individuals to 1 per 250 and an incidence of 7 cases per 100000 individuals (Bozkurt, 2016; Weintraub et al., 2017; Masarone et al., 2018). In addition, DCM comprises 60% of pediatric cardiomyopathies occurring at the highest rate during the first year of age (Towbin et al., 2006). Although different causes of DCM have been identified, many cases yet remain idiopathic. Causes of DCM are either acquired or genetic. Examples of acquired causes include infectious agents, drugs, toxins, alcohol, nutritional deficiencies, peripartum, and autoimmune, metabolic and endocrine disorders (Maron et al., 2006; Pinto et al., 2016). Although the genetic complexity of DCM is yet to be discovered, known genetic causes include mutations in more than 60 genes. The DCM-associated genes encode a variety of proteins including cytoskeletal, sarcomeric, Ca^2+^-regulating, ion-channel, desmosomal, mitochondrial, nuclear and nuclear-membrane proteins (Pérez-Serra et al., 2016). Dilated cardiomyopathy is either manifested as a predominant cardiac phenotype or associated with systemic conditions such as neuromuscular diseases (Duchenne muscular dystrophy) or syndromic diseases (Barth Syndrome) (Weintraub et al., 2017).

## Genetic Basis of Dilated Cardiomyopathy

About 35% of idiopathic DCM cases are familial and are therefore due to a genetic cause (Michels et al., 1992; McCartan et al., 2012). Familial DCM gene mutations mainly follow an autosomal dominant mode of inheritance. Nonetheless, autosomal recessive, X-linked and mitochondrial patterns of inheritance also occur but less frequently (McNally et al., 2013). The most commonly mutated gene in DCM is *TTN*, being altered in ∼25% of familial DCM cases and in 18% of sporadic cases (Herman et al., 2012; Haas et al., 2015). *TTN* encodes titin, the largest known human protein and a key component of the sarcomeres which, through its interaction with thin and thick filaments, plays a role in sarcomere assembly, passive force generation during diastole and elasticity during systole. The majority of *TTN* variants are truncating mutations (Herman et al., 2012). A study employing human induced pluripotent stem cell-derived cardiac tissue showed that certain *TTN* truncating mutations exert their pathogenicity by improper interaction with other proteins during sarcomere assembly, attenuated contractility and impaired response to stress and growth signals (Hinson et al., 2015). The *LMNA* gene, which encodes A-type lamins, is the second most commonly mutated gene in DCM, accounting for ∼6% of cases (Hershberger and Siegfried, 2011). Mutations in sarcomeric genes such as *MYH7*, *MYH6*, *MYBPC3*, *ACTC1*, *TNNT2*, and *TPM1* have also been associated with DCM, collectively being responsible for ∼5% of all cases (Kamisago et al., 2000). DCM-causing mutations in *RBM20* gene, which encodes the splicing regulator RNA binding motif protein 20 (RBM20), were first identified in 2009 (Brauch et al., 2009). The studies that followed showed that *RBM20* gene mutations occur at a rate of 3% of all DCM cases (Haas et al., 2015). Other pathogenic variants, causing a predominant cardiac DCM phenotype, have been reported in Z-disk, desmosomal and ion channel genes (Mohapatra et al., 2003; Schmitt et al., 2003; Vatta et al., 2003; Olson et al., 2005; Taylor et al., 2007).

Dilated Cardiomyopathy-associated mutations in the above genes include missense/nonsense mutations, insertions, deletions and splicing mutations (McNally et al., 2013). Variants that arise from the different mutations exert their pathogenicity via dominant negative or haploinsufficient effects of abnormal normal-sized or truncated proteins respectively. Mechanistically, the diversity of the genes involved in DCM underscore the complexity of the underlying mechanisms. Various mechanistic insights have illustrated abnormalities in protein degradation, transcriptional activity, Ca^2+^-handling and homeostasis, metabolic activity, nuclear integrity and force generation and transmission (McNally et al., 2013). Adding to the mechanistic complexity of DCM, only 30–35% of familial DCM cases follow a Mendelian mode of inheritance, suggesting a more complex multi-variant or oligogenic basis of inheritance for the remaining cases (Hershberger and Siegfried, 2011). In support of this notion, genetic screening methods have revealed the presence of nonrare variants in multiple genes for several DCM cases (Hershberger et al., 2013). Another complicating aspect is the variability of expression of the same mutation in different carriers within the same family. Variability also occurs in terms of onset, severity, progression and phenotype of disease. For instance, the 960delT mutation in the *LMNA* gene may be manifested as primary DCM, or DCM associated with either Emery-Dreifuss muscular Dystrophy (EDMD)-like or limb girdle muscular dystrophy (LGMD)-like phenotype (Brodsky et al., 2000).

## The *LMNA* Gene and Laminopathies

The *LMNA* gene encodes A-type lamins which comprise lamins A and C (lamin A/C). Lamins are type V intermediate filaments, exclusively localized to the nucleus of most differentiated cells and mesenchymal stem cells (Fisher et al., 1986; Rober et al., 1989; Ho and Lammerding, 2012). In humans, the *LMNA* gene is composed of 12 exons (Lin and Worman, 1993). Lamins A and C are produced by alternative splicing of exon 10 of the *LMNA* gene (Lin and Worman, 1993; Machiels et al., 1996). Both isoforms are identical in their first 566 amino acids after which they become different in both length and amino acid composition. While lamin C is 572 amino acids long, mature lamin A is composed of 646 amino acids. Another difference is the presence of CAAX motif at the C-terminal end of pre-lamin A which acts as a site for sequential post-translational modifications that result in cleavage of prelamin A into mature lamin A (Davies et al., 2011). Lamins A and C have a similar structural organization consisting of a short globular N terminal head domain, a central coiled-coil rod domain and a long globular C-terminal tail domain (Lin and Worman, 1993). The rod domain is highly conserved and is implicated in lamin dimerization. An immunoglobulin-like domain is also present in the tail of lamin A/C where various post-translational modifications occur (Ho and Lammerding, 2012; Burke and Stewart, 2013). Lamins assemble first by dimerizing into coiled-coiled dimers, then by forming polar polymers, through head to tail dimer arrangement, and finally by forming antiparallel nonpolar filaments (Heitlinger et al., 1992; Sasse et al., 1998; Stuurman et al., 1998). Lamins constitute the main components of the nuclear lamina and interact with proteins and DNA. Through their architectural attachment to the inner nuclear membrane, lamins provide mechanical and structural support to the nucleus. Furthermore, by acting as a platform for diverse protein interactions, they play a role in anchoring and positioning nuclear membrane proteins, regulating various signaling pathways, recruiting and sequestering transcription and DNA replication and repair factors, coupling the nucleoskeleton to the cytoskeleton and mechanotransduction. In addition, lamins bind DNA both directly and indirectly and hence play a role in chromatin organization, gene silencing and transcription (Ho and Lammerding, 2012).

The first mutation in the *LMNA* gene was identified in 1999 to be causative of EDMD, a progressive muscle weakening and wasting disorder with conduction system malfunction and DCM (Bonne et al., 1993; Bonne et al., 1999). *LMNA* gene mutations were also shown, in the same year, to be causative of DCM and conduction system disease in the absence of skeletal muscle involvement (Fatkin et al., 1999). Since then, more than 450 mutations in the *LMNA* gene have been reported^1^. The different mutations are associated with diverse diseases collectively called Laminopathies that are either manifested as tissue-specific disorders or multisystem disease (Worman and Bonne, 2007; Szeverenyi et al., 2008). While tissue-specific effects are seen in striated muscle tissue, adipose tissue or peripheral nervous tissue, multisystem effects incorporate multiple tissues and are seen in premature aging syndrome or overlapping syndromes (Cao and Hegele, 2000; Brown et al., 2001; Broers et al., 2006; Worman and Bonne, 2007). Most *LMNA* mutations (79.1%) affect striated muscle tissue, followed by adipose tissue (8.6%) and peripheral nervous tissue (0.3%). Furthermore, the percentage of *LMNA* mutations causing progeroid and overlapping syndromes is 9.3 and 10.9% respectively (Bertrand et al., 2011).

## *LMNA* Gene Mutations in Dilated Cardiomyopathy

Dilated Cardiomyopathy caused by *LMNA* mutations has the worst prognosis, highest rate of heart transplantation (Hoorntje, 2017)due to congestive heart failure and considerable risk of sudden cardiac death (Bécane et al., 2000; Taylor et al., 2003; van Berlo et al., 2005; Pérez-Serra et al., 2015). Affected individuals frequently suffer from progressive conduction system disease such as atrioventricular block, bradyarrhythmias and tachyarrhythmias and have a high chance of developing thromboembolic disorder (Fatkin et al., 1999; Arbustini et al., 2002; Fatkin and Graham, 2002; van Rijsingen et al., 2012, 2013a). Males have a worse prognosis than females, owing to frequent ventricular arrhythmias and end stage heart failure (van Rijsingen et al., 2013b). Altogether, *LMNA* mutation carriers have high disease penetrance, often presenting symptoms at an early age and having high mortality rate (Taylor et al., 2003). Furthermore, most *LMNA* mutation carriers exhibit an age-dependent penetrance, with the percentage of carriers showing a cardiac phenotype increasing from 7% under the age of 20 years to 100% above the age of 60 years (Pasotti et al., 2008).

In 2014, 165 DCM-associated mutations, based on four different databases, were identified in the *LMNA* gene (Tesson et al., 2014) (see Ref. for a detailed table of all 165 mutations). Most of these pathogenic variants were missense/nonsense mutations, some were splicing mutations, small deletions or small insertions and very few were small indel, gross deletions or gross insertions (Sébillon et al., 2003; Parks et al., 2008; Millat et al., 2009, 2011; Zimmerman et al., 2010; Narula et al., 2012; Pugh et al., 2014; Pérez-Serra et al., 2016). Since then more DCM-associated mutations were identified in the *LMNA* gene (Forleo et al., 2015; Haas et al., 2015; Pérez-Serra et al., 2015; Ambrosi et al., 2016; Hasselberg et al., 2017; Kayvanpour et al., 2017; Walsh et al., 2017). Remarkably, a recent study performed on a multicenter cohort of 77 subjects from 45 different families identified 24 novel mutations in the *LMNA* gene (Nishiuchi et al., 2017). Of these mutations, 18 were associated with DCM and were considered pathogenic (Nishiuchi et al., 2017). Most DCM-causing mutations in the *LMNA* gene occur in the head and rod domains, which comprise more than half of lamin A and two thirds of lamin C, but rarely in the tail domain. Unlike DCM, mutations linked to other laminopathies such as EDMD, familial partial lipodystrophy and Hutchinson-Gilford progeria syndrome (HGPS) commonly affect the tail domain and thus overlap with the various phosphorylation sites that are abundant in that region of the protein (Szeverenyi et al., 2008). In addition, although hot spots have been identified for some laminopathies including HGPS, mandibuloacral dysplasia and adipose tissue-specific disorders, hot spots for DCM or disorders affecting striated muscle tissue have not been recognized (Bertrand et al., 2011).

## Splicing Alterations in DCM

Several splicing factors have been associated with heart diseases including DCM (van den Hoogenhof et al., 2016). Splicing alterations in DCM include splicing factor mutations, deregulation in expression of alternative splicing isoforms and splice-site mutations. Embryonic lethality of mouse knockout models of essential splicing factors narrowed the list of splicing regulators described to cause human cardiac pathologies. Nonetheless, many of these splicing factors were shown to be embryonically lethal due to aberrant cardiogenesis. For instance, RNA binding motif protein 24 (RBM24) knockout mice die of many cardiac abnormalities and show hindered sarcomere formation. Further analysis showed that RBM24 is responsible for splicing of 64 genes many of which are important for cardiac development and sarcomere function, which goes in line with its preferential expression in striated muscle tissue (Yang et al., 2014). In addition, loss of SRSF10 (or SRp38), a ubiquitously expressed splicing factor belonging to the conserved Serine/Arginine (SR) protein family, leads to embryonic lethality due to impaired cardiogenesis. Particularly, its loss has been shown to be associated with altered expression and splicing of Ca^2+^ handling genes (Feng et al., 2009).

Another example is the ubiquitously expressed SR protein ASF/SF2 (or SFRS1), which plays a role in both constitutive and alternative splicing. As it is embryonically lethal, conditional cardiac-specific ablation of ASF/SF2 has been shown to result in DCM due to aberrant Ca^2+^ handling and excitation-contraction coupling. These effects were attributed to missplicing of several genes including Ca^2+^/calmodulin-dependent protein kinase II delta (*CamkII*δ), troponin T2 (*TNNT2*), and LIM domain binding 3 (*LDB3*) (Xu et al., 2005). SC35 (or SRSF2) is another ubiquitously expressed SR protein whose heart-specific loss results in DCM. Although a missplicing effect of SC35 was not confirmed, downregulation of ryanodine receptor 2 (RyR2) was observed in SC35-deficient hearts. This downregulation is speculated to be an effect of the nonsense-mediated decay pathway of misspliced RyR2 mRNA. In addition to its heart-specific effects, SC35 appears to be essential for embryogenesis as knockout mice die at a very early stage even before the beginning of cardiogenesis (Ding et al., 2004). In addition to SR proteins, a heterogeneous nuclear ribonuclear protein (hnRNP) family member, hnRNP U that acts both as a constitutive and alternative splicing factor, has been associated with cardiac disease. Heart specific deletion of hnRNP U was shown to be lethal during early postnatal life due to the development of severe DCM. The DCM phenotype was associated with altered alternative splicing of the Ca^2+^ handling gene *CamkII*δ (Ye et al., 2015). Dysregulation in the expression of certain splicing factors has also been shown to be associated with cardiac disease. For instance, Rbfox2 which belongs to the FOX-protein family of splicing regulators is down-regulated in heart disease. Rbfox2 regulates the alternative splicing of many genes that are related to cardiac function and its heart-specific deletion in mice develops DCM and heart failure (Wei et al., 2015).

Despite the associations of several splicing factors with cardiac pathologies, mutations in a single splicing factor, RBM20, have thus far been confirmed to cause heart disease (Brauch et al., 2009; Li et al., 2010; Refaat et al., 2012). Mutations in the *RBM20* gene have recently been shown to cause DCM (Brauch et al., 2009; Li et al., 2010; Refaat et al., 2012), putting it forward as one of the most commonly affected genes in DCM (Haas et al., 2015). In addition to being prevalent among DCM patients, *RBM20* mutations rank first for the youngest mean age of heart transplantation and are correlated with advanced disease (Brauch et al., 2009; Kayvanpour et al., 2017) (**Table 1**).

**Table 1:** A summary of the most common Rbm20 mutations.

Nucleotide Change	Protein Change	Origin	Disease association	Exon	Domain	Reference
c.1901 G > A	R634Q	Both	Causative	9	RS	Brauch et al., 2009; Li et al., 2010
c.1906 C > A	R636S	Familial	Causative	9	RS	Brauch et al., 2009
c.1907 G > A	R636H	Familial	Causative	9	RS	Brauch et al., 2009; Li et al., 2010; Wells et al., 2013
c.1909 A > G	S637G	Familial	Causative	9	RS	Brauch et al., 2009; Millat et al., 2011
c.1913 C > T	P638L	Familial	Causative	9	RS	Brauch et al., 2009; Refaat et al., 2012; Long et al., 2017; Klauke et al., 2017
c.247 C > A	L83I	Unknown	Possibly damaging by PolyPhen-2 predictions Tolerated protein function by SIFT predictions	2	Proline - rich region	Refaat et al., 2012
c.1364 C > T	S455L	Unknown	Benign by PolyPhen-2 predictions Tolerated protein function by SIFT predictions	4	-	Refaat et al., 2012
c.2109 G > C	R703S	Unknown	Probably damaging by PolyPhen-2 predictions Tolerated protein function by SIFT predictions	9	Downstream RS	Refaat et al., 2012
c.2662 G > A	D888N	Unknown	Probably damaging by PolyPhen-2 predictions Tolerated protein function by SIFT predictions	11	Glutamate - rich region	Refaat et al., 2012
c.3091 G > T	G1031^∗^	Unknown	Likely deleterious as it leads to premature stop codon	11	–	Refaat et al., 2012
c.3242 C > G	P1081R	Unknown	Probably damaging by PolyPhen-2 predictions Alter protein function by SIFT predictions	11	–	Refaat et al., 2012
c.3616 G > A	E1206K	Unknown	Possibly damaging by PolyPhen-2 predictions Alter protein function by SIFT predictions	14	–	Refaat et al., 2012
c.1661 G > A	V535I	Sporadic	No effect on Rbm20 function in splice reporter assay of titin exons	6	RRM	Li et al., 2010; Guo et al., 2012
c.1958 C > T	R634W	Familial	Unknown	9	RS	Li et al., 2010
c.1964 C > T	R636C	Familial	Causative	9	RS	Li et al., 2010; Rampersaud et al., 2011
c.2205 G > A	R716Q	Familial	Causative	9	60 residues downstream RS	Li et al., 2010
c.1903 T > G	S635A	sporadic	Loss of function of Rbm20 function in splice reporter assay of titin exons	9	RS	Guo et al., 2012
c.2062 C > T	R688^∗^	Familial	Likely Pathogenic (class 4) rare (minor allele frequency ≤ 0.1%) nonsense variant	9	downstream RS	Waldmüller et al., 2015
c.3545 G > A	R1182H	Unknown	Benign by PolyPhen-2 predictions Alter protein function by SIFT predictions Disease causing by mutation Taster predictions	13	ZnF	Zhao et al., 2015
c.2737 G > A	E913K	Familial	Causative	11	Glutamate - rich region	Beqqali et al., 2016
c.1904 C > G	S635C	Familial	Likely Pathogenic (Class 4) based on ACMG classification	9	RS	Klauke et al., 2017


*All mutations are missense except for two nonsense mutations (marked with^∗^). Most mutations occur in a 5-amino acid hotspot comprised of the residues 634, 635, 636, 637, and 638 in the RS domain of the protein. The RBM20 nucleotide position is based on the NCBI reference sequence NM_001134363.1 and amino acid positions are based on the NCBI reference sequence NP_001127835.1. RS, Arginine-Serine rich; RRM, RNA-recognition-motif, ZnF, Zinc Finger; ACMG, American College of Medical Genetics and Genomics.*

## RBM20 and Cardiac Function

**FIGURE 1:**
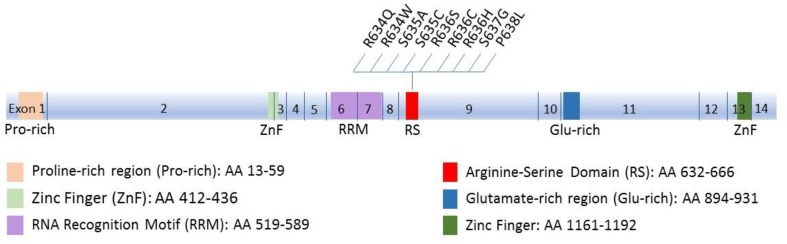
Schematic diagram of Rbm20 structure. The 14 exons and the corresponding functional domains of the protein are indicated, along with the amino acid residues spanned by each domain. All known mutations in the RS region that cluster in a five-amino acid mutation hotspot (residues 634–638) are also shown.

RBM20 is an RNA-binding protein (RBP) that regulates alternative mRNA splicing. RBM20 has one RNA recognition motif (RRM) domain in exons 6 and 7 that binds RNA, an Arginine-Serine rich (RS) domain in exon 9 that mediates interactions with other proteins, and a zinc finger domain of the U1 type (Long and Caceres, 2009; Guo et al., 2012) (**Figure 1**). RBM20 has been shown to bind to a distinct UCUU-containing RNA recognition element that is conserved between rats and humans. This binding motif is enriched within introns, such that binding of RBM20 to intronic regions flanking 3′ and 5′ splice sites represses exon splicing (Maatz et al., 2014). Many cardiac-expressed transcripts containing the UCUU motif have been shown to directly bind RBM20. The most prominent of these targets is *TTN* which encodes the protein titin (Maatz et al., 2014). In addition to *TTN*, transcripts of 17 genes (*CamkIIδ, DST, ENAH, IMMT, LDB3, LMO7, MLIP, LRRFIP1, MYH7, MYOM1, NEXN, OBSCN, PDLIM3, RTN4, RyR2, SORBS1, TNNT2*) were shown to be directly regulated by RBM20, mainly by mutually exclusive splicing (Maatz et al., 2014). Splicing regulation of mutually exclusive exons is often related to the expression of tissue-specific splice variants (Wang et al., 2008). This regulation mechanism is in accord with the tissue-specific expression of RBM20, being chiefly expressed in striated muscle with the highest amounts in cardiac muscle (Guo et al., 2012). Supporting this idea is the enrichment of the identified RBM20 targets for tissue-specific diseases associated with RBM20 mutations (DCM, hypertrophic cardiomyopathy and heart failure) according to NCBI Medical Subject Heading (MeSH) (Ho and Lammerding, 2012).

To date RBM20 has been shown to regulate the alternative splicing of 31 genes, many of which are associated with cardiomyopathies and cardiac cell biology (Guo et al., 2012). RBM20 regulates several sarcomeric genes thus influencing sarcomere structure and function. The genuine splicing target of RBM20 is titin (Li et al., 2010; Guo et al., 2012; Methawasin et al., 2014), an enormously large elastic protein that spans half the length of the sarcomere (Labeit and Kolmerer, 1995). Titin maintains the structural integrity of the sarcomere and restores it to normal length following extension and contraction (Helmes et al., 1996). Alternative splicing of titin results in many protein isoforms, the most common are N2B and N2BA isoforms (Bang et al., 2001). N2B isoform is short and rigid while N2BA isoforms are longer and more compliant (Lahmers et al., 2004). The relative ratio of short to long titin isoforms is developmentally regulated and is a determining factor of myocardial passive stiffness (Granzier and Irving, 1995; Lahmers et al., 2004). Cardiac N2BA is predominantly expressed during fetal life; however, N2B isoform becomes mainly expressed after birth (Lahmers et al., 2004; Opitz et al., 2004; Warren et al., 2004). This shift in titin isoforms is essential for proper diastolic function (Fukuda et al., 2003; Opitz et al., 2004) as N2B-enhanced passive stiffness prevents ventricular overfilling during diastole (Opitz et al., 2004). In addition, titin isoform switch is important for systolic function as N2B-increased Ca^2+^ sensitivity improves contractility during systole (Fukuda et al., 2003).

Another direct target of RBM20 is myomesin 1, a structural component of the sarcomeric M-line. Interaction of myomesin with myosin and titin is responsible for structural organization of these contractile proteins and sarcomere integrity during contraction (Agarkova and Perriard, 2005). In addition, the presence of a phosphorylation site in myomesin suggests that it responds to stretch-dependent signaling (Obermann et al., 1997). Myomesin 1 undergoes an isoform switch in a timely manner with titin (Agarkova et al., 2000). After birth, myomesin 1 isoforms that lack a molecular spring domain (EH domain) become upregulated (Agarkova et al., 2000). The myomesin 1 switch has been suggested to enhance alignment of contractile filaments and contraction efficiency (Siedner et al., 2003). Remarkably, re-expression of fetal isoforms of both titin and myomesin has been observed in DCM (Makarenko et al., 2004; Nagueh et al., 2004; Schoenauer et al., 2011). Tropomyosin 1 (TPM1), a component of thin filaments, is yet another target of RBM20 (Guo et al., 2012). Tropomyosin binds actin and mediates contraction in response to Ca^2+^ binding to troponin. Alternative splicing of TPM1 results in two striated muscle specific isoforms (TPM1α and TPM1κ) and a smooth muscle specific isoform (TBM1β). The specific role of RBM20 in these splicing events is not known. However; overexpression of the TBM1κ isoform has been observed in DCM patients and has been shown to cause systolic and diastolic dysfunction in transgenic mice (Rajan et al., 2010).

RBM20 regulates the tissue-specific splicing of Lim domain binding 3 (LDB3), a Z-line structural protein important for maintaining sarcomere integrity during contraction (Zhou et al., 2001; Guo et al., 2012; Maatz et al., 2014). RBM20 controls the inclusion of mutually exclusive exons of LDB3 that are either present in its cardiac-specific isoform (exon 4) or skeletal muscle-specific isoform (exons 5 and 6). Loss of RBM20 results in the inclusion of exons 5 and 6 rather than exon 4 (Guo et al., 2012). Moreover, mutations in exon 4 as well as in another cardiac-specific exon (exon 10) of LDB3 were identified in DCM patients (Arimura et al., 2009). Thus, loss of exon 4 due to loss of RBM20 might contribute to the development of the DCM phenotype in *Rbm20* null mice (Arimura et al., 2009; Guo et al., 2012). RBM20 is also a splicing regulator of CamkIIδ, a crucial enzyme in the heart (Guo et al., 2012). Four of the eleven isoforms described for CamkIIδ are expressed in the heart (Gray and Heller Brown, 2014). CamkIIδ isoforms phosphorylate proteins of the sarcomere and the sarcoplasmic reticulum, calcium channels and transcription factors thus affecting cell signaling, Ca^2+^ handling and gene expression (Dzhura et al., 2000; Zhang et al., 2002, 2003; Hidalgo et al., 2013). As such, splicing regulation of CamkIIδ by RBM20 can greatly affect cardiac function by modulating these processes. This is further elucidated by the association of dysregulated CamkIIδ isoforms with DCM and heart failure (Zhang et al., 2002, 2003).

## *RBM20* Gene Mutations in DCM

In an attempt to find a novel pre-clinical biomarker for DCM, genome-wide analysis of 8 families with DCM uncovered distinct heterozygous missense mutations in exon 9 of *RBM20*. These mutations segregated with the DCM phenotype, which led to the recognition of *RBM20* as a DCM-causing gene (Brauch et al., 2009). Genotype-phenotype associations linked *RBM20* mutations with aggressive DCM characterized by variable symptoms that include arrhythmias, heart failure and sudden death. Patient-derived tissues also showed variable involvement of cardiac hypertrophy and interstitial fibrosis (Brauch et al., 2009). Multiple *RBM20* mutations were identified by subsequent studies (Li et al., 2010; Refaat et al., 2012). The role of *RBM20* in DCM was revealed in rats harboring a loss of function mutation that removes the RRM, RS and zinc finger domains (exons 2–14) of *RBM20* (Guo et al., 2012). This spontaneously occurring mutation resulted in altered titin mRNA splicing. In addition to titin, alternative splicing of 30 other transcripts was shown to be altered in both rats and a DCM-patient carrying an S635A missense mutation in the RS region of RBM20. The identified RBM20-dependant genes were enriched for genes related to ion-handling, sarcomere function and cardiomyopathy (Guo et al., 2012). As most of the identified genes are key determinants of cardiac cell function and as some of them have been associated with cardiomyopathy, their missplicing is thought to be a key determinant of the DCM phenotype. This is illustrated by impairment of the Frank-Starling mechanism (FSM) as a result of expression of longer and more compliant titin isoforms in Rbm20-deficient mice (Methawasin et al., 2014). In addition to titin, missplicing of other sarcomeric proteins, such as myomesin 1, might affect their contractile function. On the other hand, splicing alterations in other RBM20 targets such as *CamkII*δ, *RyR2*, and *Cacna1c* might affect Ca^2+^ homeostasis. Indeed, Rbm20 deficiency induces a switch into larger cardiac-specific isoforms of CamkIIδ, which might compromise its normal function (Maatz et al., 2014).

In addition to altered expression of adult/fetal protein isoforms, RBM20 loss or mutation is also manifested in deregulation of tissue-specific protein isoforms or mislocalization of misspliced proteins. For instance, RyR2 and CamkIIδ aberrant splicing caused by RBM20 mutation results in the expression of mislocalized protein isoforms. RyR2 constitutes the key calcium release channel in the sarcoplasmic reticulum membrane that plays a role in excitation-contraction coupling. A 24-bp exon inclusion in RyR2 transcript causes a translocation of the corresponding protein from the ER to the intranuclear cisternae, thus deeply affecting calcium signaling (George et al., 2007). Interestingly, RyR2 transcripts containing this exon are upregulated in *Rbm20*-null rats as well as in cardiomyopathy patients (Maatz et al., 2014). Moreover, mutations affecting RyR2 function have been associated with cardiomyopathies (Tang et al., 2012). Remarkably, DCM-associated RBM20 mutation reversed the splicing of mutually exclusive exons in CamkIIδ, resulting in an isoform switch from CamkIIδB into CamkIIδA. Although both isoforms are expressed in the heart, CamkIIδB is predominantly found in the nucleus where it regulates gene expression (Zhang et al., 2002), while CamkIIδA lacks the nuclear localization signal and is mainly located in T-tubules where it plays a role in facilitation of the L-type calcium channel (LTCC) (Dzhura et al., 2000). This isoform switch has been shown, under other circumstances, to cause excitation-contraction coupling defects and proneness to tachyarrhythmia, symptoms that are also seen in DCM caused by RBM20 mutations (Xu et al., 2005).

RBM20 has also been shown to repress splicing of different targets (such as LMO7, RTN4, PDLIM3 and LDB3) in favor of their heart specific isoforms. LMO7 is a transcription factor that regulates both skeletal and cardiac muscle-related genes (Holaska et al., 2006). RBM20 represses the inclusion of exons 9 and 10 which characterize the brain specific isoform of LMO7 (Ooshio et al., 2004; Maatz et al., 2014). Although LMO7 has not been associated with DCM, expression of its brain specific isoform in the absence of RBM20 might be an important disease mechanism. Similarly, RBM20 suppresses the neuronal-specific isoform of RTN4 in favor of the heart-specific isoform (Maatz et al., 2014). RTN4 is a neurite growth inhibitor with unknown role in the heart (Huber et al., 2002). Brain-specific RTN4 isoform is weakly detected in the heart; however, it is upregulated in cases of ischemia and DCM and has also been suggested as a marker of heart failure (Bullard et al., 2008; Gramolini et al., 2008; Sarkey et al., 2011).

Most reported cardiomyopathy-related mutations in the *RBM20* gene arise in the RS region which includes a five-amino acid mutation “hotspot” within exon 9 (Brauch et al., 2009; Li et al., 2010; Millat et al., 2011; Rampersaud et al., 2011; Refaat et al., 2012; Guo et al., 2012; Wells et al., 2013; Klauke et al., 2017; Long et al., 2017). The RS region is thought to mediate protein-protein interactions (Long and Caceres, 2009). Indeed, quantitative proteomic analysis revealed that RBM20 interacts with many protein components of the U1 and U2 small nuclear ribonucleoproteins (snRNPs), which associate with pre-mRNA to form spliceosomal complex A of the spliceosome. Moreover, the RNA recognition element of RBM20 is proximal to U1 and U2 snRNP binding sites. Association of RBM20 with early-stage spliceosomal assembly but not with the catalytically active spliceosome has been suggested to stall further spliceosomal assembly beyond complex A formation, thus causing splicing repression (Maatz et al., 2014). As such, mutations in the RS region of RBM20 are expected to abrogate protein-protein interactions essential for RBM20 function as a splicing repressor. Indeed, the DCM-associated S635A mutation in the RS region of RBM20 (Guo et al., 2012) has been shown to considerably reduce interactions with 38 alternative spliceosomal factors with no effect on interactions with fundamental spliceosomal proteins. One possible explanation of this outcome is that association of RBM20 with these alternative splicing factors might be needed for the suggested spliceosomal stalling mechanism and splicing repression (Maatz et al., 2014).

In addition to protein-protein interactions, binding of RBM20 to nascent transcripts is important for its function. Indeed, mutations in exon 6 of *RBM20* were identified in idiopathic DCM patients. As these mutations localize to the RRM domain of RBM20, they are expected to disrupt its binding to mRNA (Li et al., 2010). Mice lacking the RRM domain of RBM20, by deletion of exons 6 and 7, exhibit altered titin splicing with a favored expression of more compliant titin isoforms that increased in length from heterozygous to homozygous RBM20 mutant mice. Increased titin compliance was associated with a decrease in passive stiffness and FSM which also correlated with the number of affected alleles (Methawasin et al., 2014). Along the same line, mutations in the RBM20 binding site also influence its splicing activity (Maatz et al., 2014). Although many other missense and nonsense mutations of RBM20 have been identified in DCM patients, their functional consequences have not been explored (Refaat et al., 2012; Haas et al., 2015; Zhao et al., 2015; Waldmüller et al., 2015). Yet, many of these mutations localized to novel exons of *RBM20*, including exons 2, 4, 11, 12, 13, and 14 (Refaat et al., 2012; Zhao et al., 2015; Beqqali et al., 2016).

Recently, a novel familial DCM-causing mutation (E913K) in a glutamate-rich region of RBM20, encoded by exon 11, has been studied. Although the region of the mutation is not characterized, its conservation across distinct species suggests its functional significance. This mutation was shown to cause a strong reduction in RBM20 protein levels in human cardiomyocytes, which was suggestive of compromised RBM20 protein stability. One possible mechanism that could affect protein stability is the generation of misfolded proteins and their subsequent proteasomal degradation. The outcome of reduced RBM20 protein levels was manifested in the aberrant inclusion of several exons in the spring region of titin. Missplicing of titin caused a dramatic shift from the stiff N2B isoform to the highly compliant N2BA isoform and resulted in an attenuated FSM (Beqqali et al., 2016). Notably, similar effects on titin splicing and the FSM were previously reported in a mouse model of RRM-deficient RBM20 as well as in mice lacking RBM20 (Methawasin et al., 2014).

## Deregulation in Alternative Splicing Isoform Expression

Many studies revealed, by deep sequencing and microarray analysis, sets of genes that show differential splicing between control and diseased heart; yet the mechanisms behind these alterations were not identified. In humans, splicing alterations of the sarcomeric genes, *TNNT2* (troponin T2), *TNNI3* (troponin I3), *MYH7* (myosin heavy chain 7), and *FLNC* (filamin C gamma) were observed in both DCM and hypertrophied myocardium. Interestingly, the ratio of the different splice-isoforms of each of *TNNT2*, *MYH7*, and *FLNC* served as markers that distinguished failing from non-failing heart (Kong et al., 2010). Down-regulation of the L-type voltage gated Ca^2+^ channel Cav1.2 has previously been associated with cardiac hypertrophy and heart failure (Chen et al., 2002; Goonasekera et al., 2012). Recently, a novel neonatal splice variant of Cav1.2 has been identified and was shown to be aberrantly re-expressed in adult rodent heart, upon pressure overload-induced cardiac hypertrophy, as well as in left ventricles of DCM patients. Re-expression of the identified isoform by missplicing of the Cav1.2 gene, *Cacna1c*, promoted proteasomal degradation of wild-type Cav1.2, thus explaining the reported decreased expression and activity of Cav1.2 in cardiac hypertrophy (Hu et al., 2016).

## Splice-Site Mutations

In addition to loss or dysregulation of splicing factors, splice site mutations also cause splicing alterations and disease (van den Hoogenhof et al., 2016). DCM has also been associated with splice-site mutations in its most commonly mutated gene, *TTN*. One fourth of idiopathic familial DCM cases harbor truncated titin proteins and almost 31% of mutations that generate a truncated titin protein are splice-site mutations (Herman et al., 2012). Splice site mutations in *TTN* are also thought to be responsible for HCM through the generation of a truncated titin protein which results in a reduced myocardial passive stiffness (Herman et al., 2012). However, while *TTN* truncating mutations frequently occur in DCM, they are rare in HCM (Herman et al., 2012). In addition, splice site mutations in other genes such as those encoding for lamin A/C (*LMNA*) (Parks et al., 2008), desmoplakin (DSP) (Garcia-Pavia et al., 2011) and dystrophin (*DMD*) (Obler et al., 2010) have been reported in DCM. For instance, an A > G substitution at the 3′splice site of exon 4 leads to an in-frame addition of 3 amino acids to lamin A/C protein thus causing DCM (Otomo et al., 2005).

## Lamin A/C Speckles and Splicing Factor Compartments (SFCs)

Several reports identified an association between lamin A/C and splicing factor compartments (SFCs). SFCs are 1–2 μm diameter speckles in which RNA splicing factors are concentrated. Acting as storage sites for transcription factors, SFCs are dynamic in that splicing factors are constantly recruited into and out of these compartments from and to transcription sites (Misteli et al., 1997). As such, their size changes depending on the level of transcription and mRNA splicing (Carmo-Fonseca et al., 1992; Spector, 1993; O’Keefe et al., 1994). Splicing of most nascent transcripts is simultaneous with transcription (Beyer and Osheim, 1988) and occurs on perichromatin fibrils that, in addition to being localized at the borders of SFCs, are also found throughout the nucleoplasm away from SFCs (Jackson et al., 1993; Wansink et al., 1993; Cmarko et al., 1999). In accordance with the previously proposed role of intranuclear lamins in nuclear organization, the finding that intranuclear lamin foci or speckles colocalize with RNA splicing factors in SFCs was suggestive of a structural role of lamins in SFCs (Jagatheesan et al., 1999). The findings of other studies that followed were as well implicative of a role of lamin A/C in SFC organization. For instance, the expression of terminally tagged lamin A/C resulted in depletion of lamin speckles and SFCs, which was associated with reduction in RNA polymerase II (pol II) transcription. Furthermore, lamin speckles and SFCs concomitantly became larger upon pol II transcriptional inhibition and reversibly attained their normal size upon withdrawal of inhibition (Kumaran et al., 2002). In another study published the same year, the authors showed that disruption of the nuclear organization of lamin A/C, by means of a dominant-negative mutant that lacks the N-terminal domain, also reorganizes SFCs and inhibits pol II transcription (Spann et al., 2002). Contradicting these studies, Vecerová et al. (2004) tested *Lmna*^-/-^ cells and showed that lamin A/C is non-essential for the formation and maintenance of SFCs. Nonetheless, this discrepancy between the different studies might be due to the different protein factors used to label SFCs. In addition, the expression of a truncated fragment of lamin A in *Lmna*^-/-^ cells might be sufficient to preserve lamin speckles, despite its failure to preserve the nuclear lamina (Jahn et al., 2012).

## Interaction Between laminA/C and Splicing Factors/RNA-Binding Proteins

As mentioned earlier, disruption of lamin speckles was associated with down-regulation of pol II transcription (Kumaran et al., 2002; Spann et al., 2002). This could be a direct effect of the loss of the putative interaction between lamin A/C and SFC components such as SC-35. Indeed, it has been shown that SC-35 supports pol II-dependent elongation through its interaction with cyclin-dependent kinase 9 (CDK9), a component of the positive transcription elongation factor b (P-TEFb). CDK9 phosphorylates the C-terminal domain (CTD) of pol II and results in transcriptional elongation (Lin et al., 2008). In addition to its interaction with CDK9, an interaction between SC-35 and the CTD of pol II has also been reported (Yuryev et al., 1996). Interestingly, cardiac-specific deletion of SC-35 causes DCM (Ding et al., 2004), which is also well-known to be caused by *LMNA* gene mutations (Gruenbaum et al., 2005). ASF/SF2 is another splicing factor which interacts with lamin A/C (Gallego et al., 1997; Depreux et al., 2015). ASF/SF2 is a splicing regulator of several genes that encode for cardiac proteins, such as CamkIIδ (Xu et al., 2005). As already mentioned, dysregulated CamkIIδ isoforms are associated with cardiomyopathy and heart failure (Zhang et al., 2002, 2003) and loss of ASF/SF2 in the heart tissue is responsible for DCM as well as perturbed excitation-contraction coupling (Xu et al., 2005). Therefore, the loss of association between lamin A/C and each of ASF/SF2 and SC-35 might affect their functions pertaining to striated muscle tissue which might explain the skeletal and cardiac muscle effects of most *LMNA* mutations.

A recent study identified 130 proteins that repeatedly associate with lamin A tail in C2C12 myoblasts differentiated to form myotubes. Upon functional classification of these proteins, enrichment of proteins involved in RNA splicing was noted. Furthermore, binding partners belonging to this functional category were solely found to differ between wild type lamin A and two lamin A mutants associated with EDMD (Depreux et al., 2015). Of the identified proteins in this study, 15 proteins are localized in nuclear speckles (CDC5L, DDX3X, EFTUD2, LUC7L3, NPM1, PRPF19, RNPS1, SFRS1, SFRS3, SFRS4, SRSF10, SRRM1, SRRM2, THOC4, and U2AF2) and 30 proteins in the spliceosomal complex. Furthermore, some of the identified proteins, such as ASF/SF2 (or SFRS1) and SRSF10 are localized to both compartments. As already mentioned, both alternative splicing factors have heart-specific effects despite their ubiquitous expression and their knockout in mice is embryonically lethal due to impaired cardiac development (Feng et al., 2009). Accordingly, loss of interaction between lamin A/C and these splicing factors might account for the tissue-specific effects of lamin A/C mutations.

Driven by the importance of protein interactions in core cellular processes, large-scale biochemical, proteomic and bioinformatic approaches were employed to characterize the composition of cellular protein complexes in cultured human cells (Havugimana et al., 2012). This study identified new proteins that associate with lamin A/C. Although none of the identified putative lamin A/C partners are localized to nuclear speckles, three play a role in splicing (NONO, SF3B3 and hnRNP-M). hnRNP-M belongs to the heterogeneous nuclear ribonucleoprotein (hnRNP) family of proteins. Members of this family are implicated in pre-mRNA transcription, translation, processing and transport, all of which might affect gene expression (Kim et al., 2000). They exert their effects on the fate of pre-mRNA by alternative splicing, manipulating the structure of pre-mRNA and affecting accessibility to other RNA processing factors (Martinez-Contreras et al., 2007). Remarkably, hnRNP-M has been shown to interact with cell division cycle 5-like (CDC5L) and pleiotropic regulator 1 (PLRG1) (Lleres et al., 2010), two core components of the CDC5L complex which is crucial for spliceosome assembly and function (Ajuh et al., 2000; Makarova et al., 2004). The interaction domain in hnRNP-M was shown to be essential for its role in constitutive and alternative splicing. In addition to its presence in the spliceosome, CDC5L is also present in nuclear speckles and have been shown to interact with lamin A/C (Lleres et al., 2010; Depreux et al., 2015). Other CDC5L-associated proteins that are localized to nuclear speckles and putatively interact with lamin A/C are SC-35 and ASF/SF2 (Ajuh et al., 2000).

As such, lamin A/C might regulate the pre-assembly and targeting of sub-complexes such as the CDC5L complex to the emerging spliceosomal complex, such that loss of interaction between sub-complex components and lamin A/C might influence the function of the spliceosome and gene expression. Other hnRNPs were identified by a novel approach that assesses proximity or binding to lamin A in a quite natural cellular context. This approach utilizes a biotin ligase fusion of lamin A followed by mass spectrometry of biotinylated proteins. The biotinylated hnRNPs by this procedure were hnRNP-E1, hnRNPA1, hnRNPA2B1, hnRNPA0 and hnRNPR. In addition to hnRNPs, other splicing factors that interact with and/or are proximal to lamin A, such as SF1, U2SURP, GPATCH1, DGCR14, RBM10, SUGP1, PAPOLA, TFIP11 and GTF2F2, were identified (Roux et al., 2012). Interestingly, hnRNP-E1 was shown by another study to retain its interaction with progerin, a truncated form of lamin A that causes HGPS (Zhong et al., 2005). This implies that the interaction domain might be preserved in progerin and that loss of interaction between lamin A and its interacting partners is mutation dependent, thus providing further insight into the diverse tissue-specific phenotypes associated with *LMNA* mutations (Zhong et al., 2005). In tandem with our improved understanding of DCM disease mechanisms, these insights have been enabled by the advances in next generation sequencing and transgenic models in the past two decades.

## Limitations and Future Perspectives

Not only has NGS helped in identifying mutations in a panel of DCM-causing genes, its RNA-seq application has uncovered DCM-associated defects in mRNA splicing. By means of this high-throughput technology, genetic testing has helped the diagnosis of DCM mostly through a targeted NGS panel. The benefits of such genetic diagnosis lie in determining the disease etiology, natural history and prognosis but most importantly in determining the need for family screening and the choice of a proper treatment strategy. To improve the diagnostic yield of genetic testing without increasing the chance of identifying variants of unknown significance (VUS) and incidental findings (IF), a recent study proposed a standardized stepwise exome-sequencing based approach in pediatric DCM. As this approach combines exome-based targeted analysis, copy number variation analysis and Human Phenotype Ontology (HPO) filtering, it permits heterogenous disease identification and thus personalized data analysis (Herkert et al., 2018).

Despite the progress made in identifying DCM-associated genes, further work is still needed to uncover new DCM-causing genes, and to investigate the pathogenic role and to decipher the biofunctional relevance of many of the reported mutations especially those revealed by candidate—gene approaches or identified in a small number of families. Unbiased genome wide approaches such as whole genome sequencing should be used to validate DCM-associated mutations. In addition, mutations should be replicated and validated in large cohorts. Furthermore, while most studies have focused on identifying single nucleotide variants in coding regions, other types of genomic variations such as structural variants, transposable elements insertions or variants in non-coding regions should be explored further. Studies should also tackle the role of mitochondrial variation, somatic variation, and epigenetic modifications in DCM. Confounding factors that affect the DCM phenotype in different individuals such as penetrance, effect of multiple variants, ethnic and gender differences, and environmental factors have yet to be established.

## Author Contributions

DJ and HZ contributed to the idea conception, overall review design, text mining, and interpretation of the scientific literature discussed in this review. HZ wrote the paper. DJ revised and edited the paper.

## Conflict of Interest Statement

The authors declare that the research was conducted in the absence of any commercial or financial relationships that could be construed as a potential conflict of interest.

## Footnotes

1 http://www.umd.be/LMNA/
